# Virological and serological features of acute hepatitis B in adults

**DOI:** 10.1097/MD.0000000000006088

**Published:** 2017-02-17

**Authors:** Xiaofei Du, Yali Liu, Lina Ma, Junfeng Lu, Yi Jin, Shan Ren, Zhimin He, Xinyue Chen

**Affiliations:** International Medical Department, Beijing You’an Hospital, Capital Medical University, Beijing, China.

**Keywords:** acute hepatitis B, hepatitis B virus, hepatitis B virus antigen

## Abstract

Various viral kinetics among patients with acute hepatitis B (AHB) have been observed in clinical practice. This study investigated the virological, biochemical, and serological characteristics of AHB in adults.

A total of 192 adult patients with AHB were recruited between December 2010 and January 2014. The quantification of biochemical and serologic markers for hepatitis B virus (HBV) infection was monitored from the onset.

Of the 192 patients, 113 patients were followed up. One patient died due to acute liver failure, 2 developed chronic HBV infection. Clinical recovery was observed in 110 patients; 92.7% (102/110) achieved clinical recovery within 24 weeks, and 7.3% (8/110) between 24 and 44 weeks. There were 3 different viral kinetics patterns among the patients with AHB: the clearance of HBV DNA preceded hepatitis B e antigen (HBeAg) and hepatitis B surface antigen (HBsAg), the clearance of HBeAg preceded HBV DNA and HBsAg, the clearance of HBsAg preceded HBV DNA and HBeAg.

In the absence of HBV DNA clearance within 13 weeks, the risk of development of chronic HBV infection increased. The serologic HBV markers clearance occurred between 24 and 44 weeks (6–11 months) from the onset in 8 of the AHB patients, which was longer than 6 months. Thus, AHB may be redefined as HBV DNA undetectable, HBsAg and HBeAg seroconversion within 44 weeks.

## 1 Introduction

Hepatitis B virus (HBV) infection is a serious global public health problem. According to the statistics from the World Health Organization in 2003, about 5 million people worldwide developed acute hepatitis B (AHB) annually.^[1]^ Although Chinese epidemiological studies reported that the new annual cases of AHB have decreased from about 100,000 in 2005 to 73,000 in 2010,^[2]^ HBV infection is still a serious public health problem. About 5% to 10% of the AHBs progress into chronic hepatitis B and finally into hepatic cirrhosis and liver cancer, and a few patients may develop acute liver failure^[3–6]^ which is life threatening for AHB patients. Thus, clinicians should emphasize the prevention and treatment of AHB in clinical practice. In this study, the virological, biochemical, and serological characteristics were studied in 192 adult AHB patients, to improve our understanding of clinical features of AHB, and provide some references for clinical therapy and prognosis evaluation.

## 2 Methods

Adult patients (age ≥18 years old) diagnosed with AHB were recruited from Beijing You’an Hospital between December 1, 2010 and January 31, 2014. This protocol was approved by the Institutional Review Board of You’an hospital. Written informed consent was obtained from patients, or their guardians before the study. The criteria for AHB diagnoses were based on the Chinese Management Scheme of Diagnostic and Therapy Criteria of Viral Hepatitis.^[7]^ Patients needed to meet the following criteria: no history of hepatitis B virus infection (HBsAg negative), no family history of hepatitis B infection, presence of clinical symptoms and signs of AHB, significant increase in serum transaminase with or without elevated bilirubin, positive for hepatitis B surface antigen (HBsAg), hepatitis B e antigen (HBeAg), HBV DNA, and immunoglobulin M antibody to hepatitis B core antigen positive (IgM anti-HBc). Patients with acute or chronic liver injury caused by hepatitis A, hepatitis C, hepatitis E; coinfection of hepatitis D virus, cytomegalovirus, or Epstein–Barr virus; alcoholic liver disease; and autoimmune liver diseases were excluded from the study.

The following laboratory tests were performed in this study: serum samples for liver function were tested by Olympus Au5400 biochemical analyzer and the British RANDOX reagents. Serologic markers for HBV infection were analyzed by Roche E601 automatic electrochemical luminescence instrument using the micro particle chemiluminescence method and Roche kits (lower limit of detection of HBsAg was 0.05 IU/mL), according to the manufacturer's instructions. Quantitation of HBV DNA was performed by polymerase chain reaction using the Roche Cobas/Taqman Real-Time PCR 2.0 System, according to the manufacturer's instructions (lower limit of detection was 20 IU/mL). HBV S region sequences were amplified by nested PCR to test HBV genotype. PCR primers were provided by Beijing Shenggong Technology Co Ltd, and sequencing was performed by Beijing Bomad Biological Co Ltd. Biochemical and serological markers were tested weekly.

Statistical analyses were conducted using SPSS version 21.0 for Windows (IBM Inc, Chicago, IL). Data with normal distribution are expressed as mean ± standard deviation, *t* test (for 2 groups) or 1-way analysis of variance (for more than 2 groups) were used. Categorical data were expressed as median (interquartile range) and compared using the *χ*^2^ test. A 2-sided value of *P* < 0.05 was considered statistically significant.

## 3 Results

A total of 192 patients with AHB were recruited in this study. All patients were Han Chinese, 126 (65.6%) males and 66 (34.4%) females with the male-to-female ratio of 1.9:1. The mean age of onset was 41.7 ± 12.9 years (range: 18–80 years). The median HBV DNA was 7470 IU/mL (Table 1). The majority of patients were aged 26 to 65 years (n = 163; 84.9%). The routes of transmission are shown in Table 2: 116 patients were unknown; while 76 patients had a known route of transmission, 49 patients (49/76, 64.5%) were through sexual transmission. Nonaseptic operations and professional exposure were 32.9% (25/76) and 2.6% (2/76) respectively.

**Table 1:**
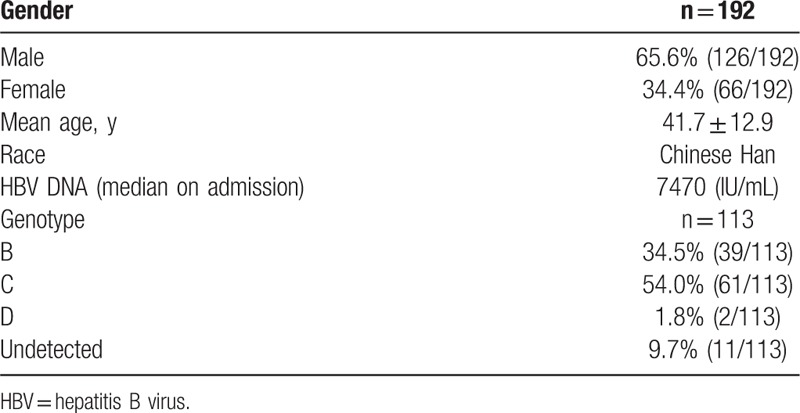
Characteristics of the patient population with acute hepatitis B.

**Table 2:**
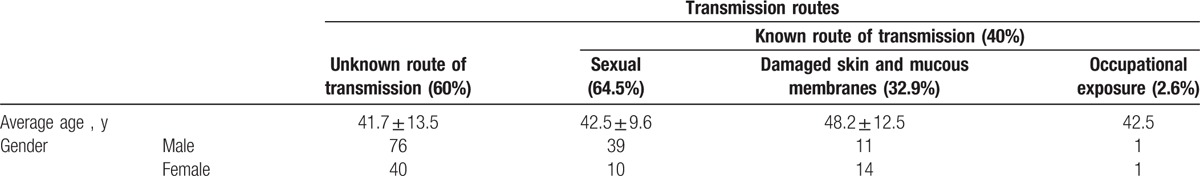
Transmission routes.

The major clinical manifestations included fatigue (70%), gastrointestinal symptoms (poor appetite, nausea, vomiting; 80%), yellow urine (68.5%), and fever (8%). A few patients had joint pain. Gastrointestinal symptoms were relieved within 2 weeks in 80.3% of patients (154/192). On admission, clinical examination showed icteric skin and sclera (90%), liver pain on percussion (73%), and mild hepatomegaly (30%). Symptomatic supportive treatments were applied to all the patients, including close monitoring of vital signs, fluid status, nutritional status, electrolytes, and liver function. For the patients with acute liver failure, entecavir was used for antiviral treatment, antibiotics were used for infection and treatment of hepatic encephalopathy, and terlipressin and albumin for hepatorenal syndrome. Symptoms resolved within 2 weeks in 80% of the patients, and within 4 weeks in 95% of patients.

Of the 192 patients, 113 were followed up (79 lost to follow-up). One patient died due to acute liver failure, 2 progressed to chronic HBV infection, and 110 patients reached clinical recovery. For HBV genotype testing, 102 of 113 patients were sequenced successfully. Genotype distribution in these patients was as follows: Genotype B accounted for 34.5% (39/113), genotype C for 54.0% (61/113), genotype D for 1.8% (2/113) and 9.7% (11/113) were undetected (Table 1). HBV DNA, serologic markers for HBV infection, and clinical outcomes were analyzed for the 110 patients achieving clinical recovery. In this study, the time of AHB recovery was defined as the interval between the presence of evident clinical symptoms and clinical recovery (HBV DNA clearance with HBsAg and HBeAg seroconversion). Based on this definition, the median time of recovery was 8 weeks (range: 1–44 weeks). One hundred two patients (102/110, 92.7%) achieved clinical recovery within 24 weeks, and the other 8 patients (8/110, 7.3%) achieved clinical recovery between 24 and 44 weeks. The length of time from the onset to the change in HBV serologic markers is shown in Table 3. The median HBeAg clearance time was 3 weeks, and 90% of the patients achieved HBeAg seroconversion in 8 weeks. The median HBsAg clearance time was 6 weeks, and 90% of the patients achieved HBsAg seroconversion in 19 weeks.

**Table 3:**
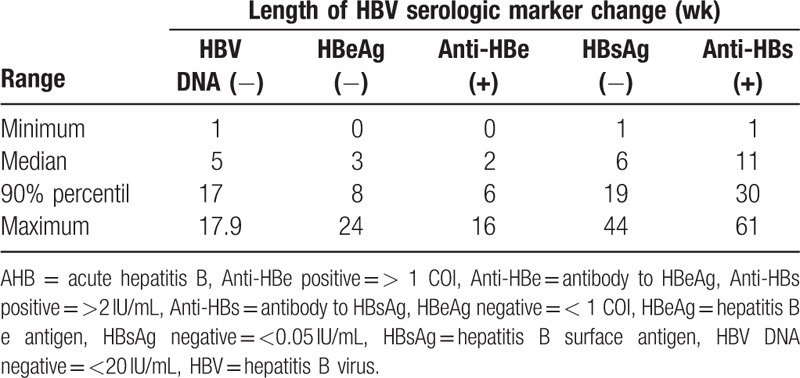
Time of HBV serologic markers change for AHB infection.

The biochemical parameters of the 110 patients are shown in Table 4. One hundred five patients (105/110, 95.5%) had jaundice hepatitis. Alanine aminotransferase (ALT) was more than 35 times the upper limit of normal (1419.3 U/L), and total bilirubin (TBiL) was higher than 111.4 μmol/L in 50% of patients. ALT, aminotransferase (AST), and TBiL returned to normal in 50% patients within 5 to 6 weeks.

**Table 4:**
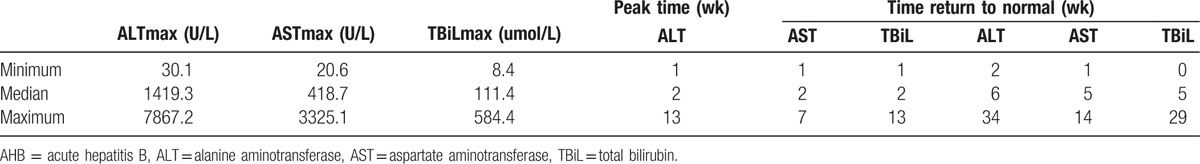
AHB serum liver biochemistries (n = 110).

**Figure 1:**
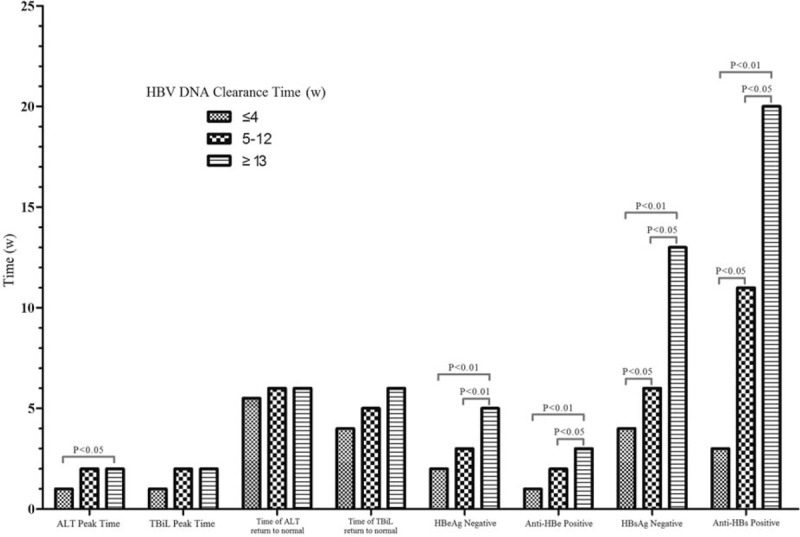
The clearance time of HBV DNA did not affect the levels of ALT and TBiL as well as the time of they becoming normal, but significantly affected the time of HBeAg and HBsAg seroconversion. ALT = alanine aminotransferase, HBeAg = hepatitis B e antigen, HBsAg = hepatitis B surface antigen, HBV = hepatitis B virus, TBiL = total bilirubin.

According to the time needed for HBV DNA clearance, patients were divided into ≤4 weeks, 5 to 12 weeks, and ≥13 weeks. As shown in Fig. 1, the length of HBV DNA clearance did not affect the length of ALT and TBiL recovery, but significantly affected the time of HBeAg and HBsAg seroconversion. In addition, the length of HBV DNA clearance had an influence on the overall time of clinical recovery. All the patients with undetectable HBV DNA (87/87, 100%) within 12 weeks (46 patients in 4 weeks and 41 patients in 5–12 weeks) achieved clinical recovery within 24 weeks, but only 65.2% (15/23) of those with undetectable HBV DNA after 12 weeks achieved clinical recovery within 24 weeks. The remaining patients (8/23, 34.8%) achieved clinical recovery within 24 to 44 weeks (Table 5). In addition, HBV DNA was still present at 76 weeks from the onset, for 2 genotype C AHB patients who progressed to chronic HBV infection.

**Table 5:**
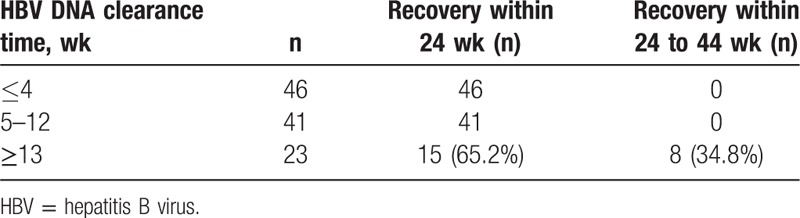
Effect of HBV DNA clearance time on disease course.

In this study, according to the sequence of HBV DNA, HBeAg, and HBsAg clearance, there were 3 different viral kinetic patterns among the patients with acute hepatitis B: HBV DNA undetectable (25.5%, 28/110): the clearance of HBV DNA preceded HBeAg and HBsAg. HBV DNA undetectable, then HBeAg seroconversion followed by HBsAg seroconversion (17.3%, 19/110); HBV DNA undetectable, then HBsAg seroconversion followed by HBeAg seroconversion (2.7%, 3/110); HBV DNA undetectable accompanied by HBeAg and HBsAg seroconversion (5.5%, 6/110). HBeAg undetectable (71.8%, 79/110): the clearance of HBeAg preceded HBV DNA and HBsAg. HBeAg seroconversion, then HBV DNA undetectable followed HBsAg seroconversion (41.8%, 46/110); HBeAg seroconversion, then HBsAg seroconversion followed HBV DNA undetectable (30%, 33/110). HBsAg undetectable (2.7%, 3/110): the clearance of HBsAg preceded HBV DNA and HBeAg. HBsAg seroconversion, then HBV DNA undetectable, and HBeAg seroconversion (0.9%, 1/110); HBsAg seroconversion, then HBeAg seroconversion, and HBV DNA undetectable (1.8%, 2/110).

## 4 Discussion

In our study, the number of male patients was higher than that of females, and the majority of adult AHB patients were aged 26 to 45 years, similar to previous studies.^[1,8,9]^ The incidence of AHB has declined since the introduction of hepatitis B vaccination in China,^[2]^ from 7.5/100000 people in 2005 to 5.6/100000 people in 2010; the incidence of AHB is lower in children younger than 15 years of age. However, the incidence of AHB in adults has not significantly declined and even has a rising trend in some regions. Our results were consistent with the overall epidemiological characteristics of AHB and show that young adults have a high risk for AHB.

In the present study, 60% (115/192) of patients could not explain the route of transmission, as has been reported in other Chinese studies.^[10]^ In the patients with known transmission routes, sexual transmission was the main cause of AHB in males, which is consistent with previously reported studies.^[10–12]^ The numbers of males and females were comparable with respect to other routes of transmissions. In Western countries, the major routes of transmission are sexual contact and intravenous drug abuse.^[13]^ Our results showed patients aged 26 to 65 years (especially male patients) had a high risk for AHB. Thus, screening for serologic markers for HBV infection is recommended in this population, and vaccination may be administered if necessary. If the sexual partner has chronic HBV infection, both active immunization by vaccination and other preventive measures (such as the use of a condom) are recommended.

In the present study, 95.5% of patients presented with jaundice and 62.7% of patients with moderate to severe jaundice, which was rarely reported in previous studies.^[14,15]^ One AHB patient in our study died due to acute liver failure. Therefore, it is important to closely monitor the disease progression in AHB patients with severe jaundice; oral nucleoside antiviral drugs are recommended to improve the prognosis.

There is still controversy about the effects of serum viral load on the course of the disease. Some studies^[16,17]^ report that an HBV DNA level >10^6^ copies/mL at 8 weeks is associated with disease chronicity (the definition of chronicity being HBsAg was still present after 24 weeks from the onset, regardless of the liver function recovery). Our study revealed that the time of ALT peak, HBeAg seroconversion, and HBsAg seroconversion was shorter in patients whose HBV DNA was undetectable ≤4 weeks compared with ≥13 weeks (*P* <0.01). It is notable that 34.8% (8/23) of patients whose HBV DNA clearance occurred at ≥13 weeks achieved clinical recovery in 24 to 44 weeks. Hence, the time of clinical recovery is in accordance with the time of HBV DNA clearance. The disease may become chronic if HBV DNA clearance occurs slowly. HBV DNA was still present in 2 patients with AHB and HBsAg positivity at >44 weeks who were followed up for 76 weeks. Therefore, for patients with HBV DNA detectable for more than 44 weeks, physicians need to follow them carefully and appropriate antiviral treatment may be administered to prevent the chronicity of HBV infection. At present, it is generally accepted that AHB in only 5% to 10% of patients will progress into chronic hepatitis, and thus antiviral treatment is not recommended for AHB.^[18]^ However, there is a large population susceptible to hepatitis B in China and the number of AHB patients developing chronic disease will be very large even if only 5% of AHB patients have the possibility of developing chronic disease. Therefore, timely antiviral treatment may be administered according to the disease condition, such as the presence of trend to chronicity.^[6,19–23]^ In this study, 7.3% (8/110) of patients achieved clinical recovery between 24 and 44 weeks, and 2 patients developed chronic disease. We speculate that the time point of 44 weeks after onset seems to be more suitable to define the clinical recovery or disease chronicity in AHB patients.

Our study showed 71.8% of AHB patients had HBeAg seroconversion first, and HBV DNA was undetectable. HBeAg is a structural protein of HBV and plays an important role in the regulation of immune function. It has been used as a marker indicating active viral replication. In this study, 50% of patients achieved HBeAg seroconversion within 3 weeks, suggesting that immunity is activated after HBV infection to clear viruses in most AHB adults. In addition, another 3 patients presented HBsAg seroconversion before the HBV DNA became undetectable, which was different from the findings reported in previous studies.^[3,11]^ The nonclassical seroconversion mode of serologic markers for HBV infection accounted for 74.5% of the patients, while classic mode for only 25.5%. In the classical mode, there were still differences compared with previously reports: 2.7% (3/110) of the patients cleared HBV DNA first, followed by HBsAg seroconversion and then HBeAg seroconversion. HBV DNA clearance, HBeAg and HBsAg seroconversion occurred simultaneously in 5.5% (6/110) of the patients. The nonclassic mode of seroconversion was also reported in our previous study on the antiviral therapy in chronic hepatitis B patients.^[24]^ In that study, we speculated that it was related to interferon therapy. However, this mode still exists in AHB patients without antiviral therapy. This may be explained as follows: the HBV DNA, HBeAg, and HBsAg are synthesized in different ways. HBeAg and HBsAg are encoded by different mRNAs. The precursor mRNA genome serves as a template for the synthesis of new HBV DNA and guides the synthesis of viral proteins such as HBV polymerase, core protein and e protein. Three small mRNA transcripts can be used to synthesize other viral proteins in the endoplasmic reticulum of the host, forming complete viral HBsAg. The classical mode of serologic markers for HBV infection seroconversion is determined based on the outdated detection reagents. Detection using outdated reagents has poor specificity and sensitivity, and may fail to reflect actual changes. Based on the different seroconversion modes of serologic markers for HBV infection and HBV DNA, we speculate that it is necessary to redefine the “clinical recovery” of AHB. The limitations of this study include incomplete data due to the loss to follow-up of 79 subjects. The sample size was relatively small, and this was a single-center study.

## 5 Conclusions

The clinical recovery of AHB should be defined as HBsAg seroconversion with HBV DNA, and HBeAg seroconversion instead of HBsAg seroconversion alone. In addition, the time point of 44 weeks after onset may be a better cut point to define the clinical recovery or disease chronicity in AHB patients. Due to the limitations mentioned above, the conclusions need to be further confirmed by multicenter, large sample size studies.
